# Astrogliosis Associated With Behavioral Abnormality in a Non-anaphylactic Mouse Model of Cow's Milk Allergy

**DOI:** 10.3389/fncel.2019.00320

**Published:** 2019-07-16

**Authors:** Nicholas A. Smith, Danielle L. Germundson, Colin K. Combs, Lane P. Vendsel, Kumi Nagamoto-Combs

**Affiliations:** ^1^Department of Pathology, University of North Dakota School of Medicine and Health Sciences, Grand Forks, ND, United States; ^2^Department of Biomedical Sciences, University of North Dakota School of Medicine and Health Sciences, Grand Forks, ND, United States

**Keywords:** food allergy, astrocyte, anxiety, depression, intestine, occludin, tumor necrosis factor, neuroinflammation

## Abstract

Etiology of neuropsychiatric disorders is complex, involving multiple factors that can affect the type and severity of symptoms. Although precise causes are far from being identified, allergy or other forms of hypersensitivity to dietary ingredients have been implicated in triggering or worsening of behavioral and emotional symptoms, especially in patients suffering from depression, anxiety, attention-deficit hyperactivity, and/or autism. Among such ingredients, cow's milk, along with wheat gluten, is commonly suspected. However, the contributory role of cow's milk in these disorders has not been elucidated due to insufficient pathophysiological evidence. In the present study, we therefore investigated neuroinflammatory changes that are associated with behavioral abnormality using a non-anaphylactic mouse model of cow's milk allergy (CMA). Male and female C57BL/6J mice were subjected to a 5-week oral sensitization procedure without or with a major milk allergen, beta-lactoglobulin (BLG). All mice were then later challenged with BLG, and their anxiety- and depression-associated behaviors were subsequently assessed during the 6th and 7th weeks. We found that BLG-sensitized male mice exhibited significantly increased anxiety- and depression-like behavior, although they did not display anaphylactic reactions when challenged with BLG. Female behavior was not noticeably affected by BLG sensitization. Upon examination of the small intestines, reduced immunoreactivity to occludin was detected in the ileal mucosa of BLG-sensitized mice although the transcriptional expression of this tight-junction protein was not significantly altered when measured by quantitative RT-PCR. On the other hand, the expression of tumor necrosis factor alpha (TNFα) in the ileal mucosa was significantly elevated in BLG-sensitized mice, suggesting the sensitization had resulted in intestinal inflammation. Inflammatory responses were also detected in the brain of BLG-sensitized mice, determined by the hypertrophic morphology of GFAP-immunoreactive astrocytes. These reactive astrocytes were particularly evident near the blood vessels in the midbrain region, resembling the perivascular barrier previously reported by others in experimental autoimmune encephalitis (EAE) mouse models. Interestingly, increased levels of COX-2 and TNFα were also found in this region. Taken together, our results demonstrated that BLG sensitization elicits inflammatory responses in the intestine and brain without overt anaphylactic signs of milk allergy, signifying food allergy as a potential pathogenic factor of neuropsychiatric disorders.

## Introduction

Behavioral and emotional disorders, such as anxiety, depression, attention-deficit hyperactivity disorder (ADHD), obsessive-compulsive disorder, and autism, are major mental health problems that could severely affect quality of life. In the United States alone, ~20% of adolescents and adults are reported to have experienced mental disorders in 2016^1^. The actual number of people who suffer from these conditions is expected to be greater than reported, considering that these disorders often go undiagnosed due to unwillingness of patients to disclose their conditions or failure of their caregivers to recognize the symptoms (Glazier et al., 2015; Hirschtritt et al., 2017; Klik et al., 2018). Even among diagnosed patients who seek treatments, some are resistant to conventional pharmacological and psychotherapeutic interventions, requiring increased dosage of medications and/or more aggressive treatments such as electroconvulsive therapy and neurostimulation (Al-Harbi, 2012; Hirschtritt et al., 2017). Nonetheless, not all patients benefit from these treatments, signifying the need for alternative intervention approaches for these debilitating conditions.

Interestingly, certain dietary items have been long suspected to trigger or exacerbate emotional and behavioral symptoms (Crayton, 1986), suggesting a potential role of food allergy/hypersensitivity (FAH) in the etiology of neuropsychiatric conditions. While many clinical cohort studies have reported that significant behavioral comorbidities exist among individuals with FAH (Addolorato et al., 1998; Parker and Watkins, 2002; Costa-Pinto and Basso, 2012; Shanahan et al., 2014; Ferro et al., 2016), the contributory role of diet in neuropsychiatric disorders has been controversial due to insufficient pathophysiological evidence and inconsistent results across studies. In order to determine the causative role of FAH in behavioral changes without genetic, dietary, and environmental variables commonly associated with human cohorts, we utilized a mouse model of cow's milk allergy (CMA) and examined behavioral changes and pathophysiology in the gut and brain mediated by FAH.

To observe CMA-mediated changes in typical innate activities of mice, a non-anaphylactic mouse model of CMA was previously established by orally sensitizing the C57BL/6J strain of mice with a whey protein (WP) mixture and cholera toxin (CT) as an adjuvant (Germundson et al., 2018). These sensitized mice generally exhibited mild to no anaphylaxis upon WP challenge, allowing a series of behavioral assessments to be performed the next day. In this study, we limited the allergen to β-lactoglobulin (BLG; Bos d 5), in order to isolate the effect of this major whey allergen, which is absent in human breast milk (Malacarne et al., 2002). We report that BLG-sensitized male mice displayed anxiety- and depression-like behavior similar to the mice sensitized with the WP mixture. Moreover, we found astrocytic hypertrophy in the ventral midbrain of the BLG-sensitized brain, particularly near the blood vessels, resembling the perivascular “barriers” or “cuffs” described in mouse models of experimental autoimmune encephalitis (EAE) (Voskuhl et al., 2009; Sofroniew and Vinters, 2010). These results indicated that oral BLG sensitization of otherwise healthy mice results in region-specific perivascular astrogliosis, likely modifying the functional property of the blood brain barrier.

## Materials and Methods

### Mice

C57BL/6J mice were purchased from The Jackson Laboratories (Bar Harbor, ME, USA) and housed in a pathogen-free room at the University of North Dakota animal facility under 12-h light/12-h dark cycle. Male and female pups were weaned at 3 weeks old and placed on a whey-free rodent diet (Teklad 2018, Envigo, Indianapolis, IN, USA) with *ad libitum* access to ultra-filtered water. Mice were weighed weekly and their health and growth were monitored throughout the experiment. All procedures involving mice were approved by the University of North Dakota Institutional Animal Care and Use Committee.

### BLG Sensitization Procedure

**Figure 1:**
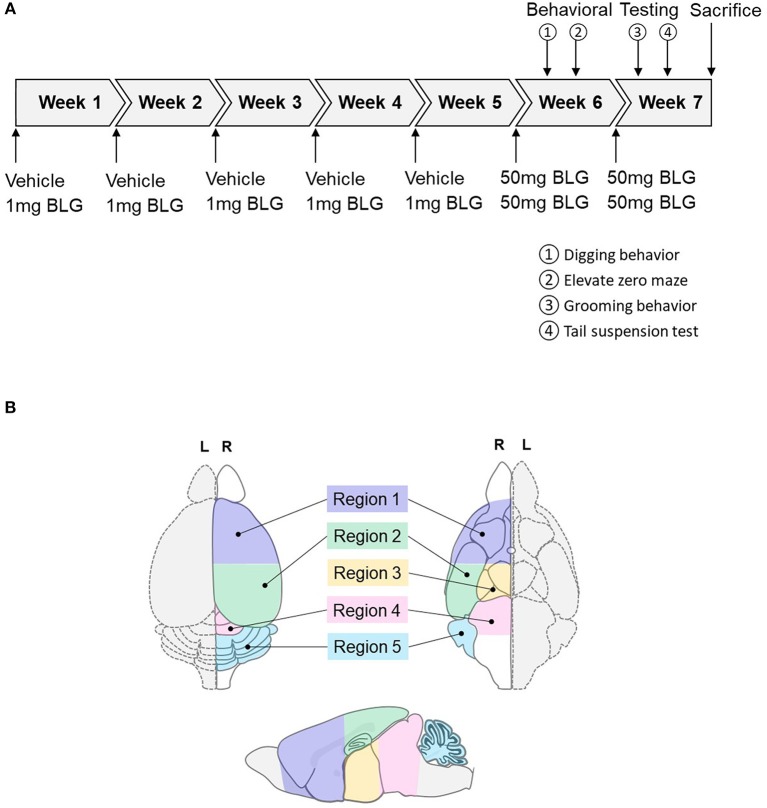
Schematics of the experimental timeline and the regions of the brain analyzed in the study. **(A)** Starting at 4-weeks of age, mice were given a weekly oral administration of 200 μL vehicle (carbonate/bicarbonate buffer containing 10 μg CT, pH 9.6) with or without 1 mg BLG for 5 weeks. In the 6th and 7th weeks, all mice were challenged with 50 mg BLG in carbonate/bicarbonate buffer (without CT), and their behavior was subsequently tested at 1- and 2-day post-challenge. One day after the last behavior test in Week 7, mice were sacrificed, and blood and tissue samples were harvested. **(B)** Diagrams depicting the dorsal (left), ventral (right), and sagittal (bottom) views of a mouse brain. Upon collection of brain samples, the left hemisphere (L, dotted outline) was immersion-fixed in 4% PFA while the right hemisphere (R, solid outline) was subdivided into the following five regions: Region 1, rostral brain including prefrontal and frontal cortices and underlying subcortical structures (e.g., striatum); Region 2, parietotemporal cortices and the hippocampus; Region 3, the thalamus and hypothalamus, Region 4, the midbrain; and Region 5, the cerebellum.

At 3-weeks of age, mice were randomly divided into sex-matched sham and BLG-treatment groups. One week later, sensitization was carried out for 5 weeks as described previously with modifications (Germundson et al., 2018). In brief, BLG-sensitized mice were given a weekly oral gavage dose of 1 mg BLG (#L0130, MilliporeSigma, Burlington, MA, U.S.A.) in 200 μL of a sodium carbonate/bicarbonate-buffered vehicle [pH 9.6] containing 10 μg per dose of cholera toxin (CT; #100B, List biological Laboratories, Inc., Campbell, CA, USA) as the adjuvant. The sham mice received the CT-containing vehicle without BLG. On the 6th week, all mice were orally challenged with 50 mg BLG in 200 μL carbonate/bicarbonate buffer. Thirty minutes after the challenge, presence or absence of hypersensitivity reactions, such as scratching of face and ears, perioral swelling, decreased mobility, respiratory distress, and/or lethargy, were noted. Behavioral tests were subsequently performed during the next 2 days. Mice were challenged one more time with 50 mg of BLG on the 7th week followed by two more days of behavioral tests and sacrificed the following day. A schematic depicting the sensitization and challenge schedule is shown in Figure 1A.

### Behavioral Testing

Behavior of mice was evaluated over the course of 2 days after each of the two BLG challenges in Week 6 and 7 (Figure 1A). One day after the first challenge in Week 6, innate digging behavior was assessed by placing each mouse in an enclosure (24.8-cm width × 38.7-cm depth × 29.2-cm height containing 5-cm thickness of clean corncob bedding. Mice were acclimated for 5 min and allowed to freely explore the cage for 15 min thereafter. Their activities were recorded on a digital camera and the occurrence of digging activity was counted from the video recordings by two observers who were blinded to the experimental conditions of the mice. On the second day after the challenge in Week 6, anxiety-like behavior was observed using an elevated zero maze (EZM; Stoelting Co., Wood Dale, IL, USA). Activity of each mouse on the EZM was recorded for 5 min and the amount of time mice spent in the open and closed zones of the maze and the number of entries to each of the zones were recorded.

One day after the second BLG challenge in Week 7, mice were individually placed in empty cages and their grooming behavior was video-recorded for 10 min after 5 min of acclimation. As with the scoring of digging behavior, the frequency of grooming behavior was counted by two blinded observers from the video recordings. The presence or absence of grooming activity was monitored by giving 1 or 0 point, respectively, during each of 60 × 10-s intervals and the sum of the points for the testing period (10 min) was considered the grooming frequency of the mice. On the second day after the second challenge, a tail suspension test (TST) was performed based on a previously described protocol (Can et al., 2012). Briefly, mice were suspended by their tail from a horizontal bar with a piece of laboratory tape so that their nose was ~ 30 cm above the base of the bar support. Because the C57BL/6 strain is known for their tendency to climb their tail (Mayorga and Lucki, 2001), a piece of plastic tubing was used to maintain the mice in the suspended position. Their attempts to escape from the position were video-recorded for 6 min, and the frequency and length of immobility as well as the latency to the first immobile episode were compared between groups as indications of depression-like behavior.

All behavior testing was performed at the University of North Dakota Behavioral Research Core Facility. The ANY-maze software (Stoelting, Co.) was used to establish all test parameters, to control video recordings, and to compute the results.

### Tissue Collection

Mice were sacrificed 1 day after the behavioral tests in Week 7 via CO_2_ asphyxiation followed by cardiac puncture and exsanguination. Terminal blood was collected before transcardiac perfusion with phosphate-buffered saline (PBS). The brain from each mouse was carefully removed and bisected sagitally. The left hemisphere was immersion fixed in 4% paraformaldehyde for histological analysis and the right hemisphere further dissected into 5 regions. Region 1 contained the prefrontal and frontal cortices and underlying subcortical structures including the striatum; Region 2 comprised the parietotemporal cortices and underlying hippocampus; Region 3 contained the thalamus and hypothalamus; Region 4 included the midbrain and rostral brainstem; and Region 5 was the cerebellum (Figure 1B). Each of these regional samples was immediately frozen and stored at −80°C until used for western blot and ELISA analyses. The ileum portion of the small intestine was also dissected and fixed for immunohistological staining or frozen stored for RNA isolation for RT-qPCR analysis.

### Enzyme-Linked Immunosorbent Assay (ELISA) for the Detection of BLG-Specific IgE/IgG1 and TNFα

The amount of antigen-specific immunoglobulin E (IgE) and G1 (IgG1) antibodies present in sera was quantified using ELISA as described previously with modifications (Germundson et al., 2018). Briefly, the wells of the ELISA plate were coated with 20 μg/mL BLG in a 100 mM sodium carbonate/bicarbonate buffer overnight at 4°C, washed thoroughly and blocked with PBS containing 0.05% Tween-20 and fetal bovine serum (Assay Buffer, eBioscience ELISA Support Pack Plus # BMS414, Thermo Fisher Scientific, Waltham, MA, USA). Sera isolated from the terminal blood of mice were diluted 1:1 for IgE or 1:50 for IgG1 detection with Assay Buffer before adding to the antigen-coated wells. The plate was incubated for 12 h at 4°C and BLG-specific IgE was detected with biotinylated anti-mouse IgE (used at 1:1,000, #13-5992-81, ThermoFisher Scientific) or anti-mouse IgG1 (used at 1:1,000, #13-4015-82, ThermoFisher Scientific) followed by avidin-HRP and TMB (3,3′,5,5′-tetramethylbenzidine) according to the manufacturer's instructions. The plate was read at 450 nm on a Biotek ELx 800 microplate reader using Gen5 v3.02 software (Biotek Instruments, Winooski, VT, USA).

The amount of tumor necrosis factor alpha (TNFα) in the midbrain samples (Region 4) was quantified using TNFα DuoSet® ELISA (#DY410, R&D Systems, Minneapolis, MN, USA) according to the manufacturer's protocol. Briefly, an ELISA plate (#2580, Corning EIA/RIA 8-Well Strips) was coated with TNFα capture antibody overnight at room temperature. After washing and blocking the wells, each protein extract from Region 4 (200 ng/100 μL/well) was placed in duplicates and incubated for 2 h at room temperature. TNFα in the samples were visualized by sequentially incubating the wells with the detection antibody and streptavidin-HRP. The substrate reaction was allowed to occur for 20 min before termination, and the plate was read as described above. The duplicate values from each sample were averaged and TNFα concentration was calculated from the standard curve.

### Immunohistochemistry

Tissues collected for histology were immediately immersion-fixed in 4% paraformaldehyde. Fixed ileal and brain tissues were embedded in gelatin and frozen sectioned at 14 and 40 μm, respectively. For immunostaining, tissue sections were treated with 0.3% peroxidase and blocked in PBS containing 0.5% bovine serum albumin and 5% normal goat serum (#16210072, ThermoFisher Scientific), and incubated with a primary antibody against rabbit anti-mouse occludin (used at 1:100, #711500, ThermoFisher Scientific), glial fibrillary acidic protein (GFAP; used at 1:1,000, #12389S, Cell Signaling Technology, Boston, MA, USA) or Iba1 (used at 1:1000, #019-19741, Wako Chemicals, Richmond, VA) for 24–48 h at 4°C. Tissues were subsequently incubated in anti-rabbit IgG (used at 1:2,000, #PK-6101, Vector Laboratories, Burlingame, CA, USA) or mouse-adsorbed rabbit anti-rat IgG (used at 1:100, #BA-4001, Vector Labs) antibody. Immunoreactivity was visualized with Vector Elite ABC kit (#PK-6101, Vector Labs) with VIP as the chromogen (#SK-4600, Vector Labs).

### Reverse Transcriptase Quantitative Polymerase Chain Reaction (RT-qPCR)

Ileal tissue samples were homogenized in TRIzol solution (#15596018, Thermo Fisher Scientific) using 5-mm stainless steel beads in a Bullet Blender Storm 24 (Next Advance, Troy, NY, USA) set to speed 6 for 3 min with 30-s intervals. Total RNA was extracted and purified by ethanol precipitation according to the manufacturer's instructions. The amount of RNA was quantified using Nanodrop One (Thermo Fisher Scientific), and 1 μg of total RNA was used to synthesize cDNA libraries with an iScript™ cDNA Synthesis Kit (#1708891, Bio-Rad Laboratories, Hercules, CA, USA) by priming at 25°C for 5 min, reverse transcription at 46°C for 20 min, and inactivation at 95°C for 1 min. Quantitative PCR reactions were performed with 100 ng cDNA and specific primer sets for murine TNFα (*Tnf*α*;* qMmuCED0004141, Bio-Rad), occludin (*Ocln*; fwd: 5′-AAAGCAAGTTAAGGGATCTG-3′; Rev: 5′-TGGCATCTCTCTAAGGTTTC-3′, MilliporeSigma), or glyceraldehyde 3-phosphate dehydrogenase (*Gapdh*; qMmuCED0027497, Bio-Rad) using iTaq™ Universal SYBR® Green Supermix (#1725120, Bio-Rad) on a C1000 Touch Thermo Cycler (Bio-Rad) for 40 cycles (denaturing at 95°C for 15 s, annealing at 60°C for 30 s, and extension at 72°C for 30 s). Resulting Cq values were calculated using Bio-Rad CFX Manager Software version 3.1.

### Western Blot Analysis

Total proteins from each isolated region of the right brain hemisphere were extracted in RIPA buffer (20 mM Tris, pH 7.4, 150 mM NaCl, 1 mM Na_3_VO_4_ 10 mM sodium fluoride, 1 mM EDTA, 1 mM EGTA, 0.2 mM phenylmethylsulfonyl fluoride, 1% Triton X-100, 0.1% SDS, and 0.5% deoxycholate) and quantified using the Bradford method (Bradford, 1976). Western blotting was carried out as described previously (Nagamoto-Combs et al., 2014) with 25 μg of protein samples resolved on 15% SDS-polyacrylamide gels. Resolved proteins were transferred onto PVDF membranes and detected with a primary antibody against GFAP (used at 1:1,000, #12389S, Cell Signaling Technology), cyclooxygenase 2 (COX-2; used at 1:1,000, #sc-1745, Santa Cruz Biotechnology, Santa Cruz, CA, USA) or GAPDH (used at 1:1,000, sc-32233, Santa Cruz Biotechnology) overnight at 4°C or 2 h at room temperature with gentle rocking. The membranes were subsequently incubated in an appropriate HRP-conjugated secondary antibody (Santa Cruz Biotechnology). Target proteins were visualized using Amersham ECL Prime Western Blotting Detection Reagent (#RPN2232, GE Healthcare, Pittsburgh, PA, USA) on an Aplegen Omega Lum G Gel Documentation System (Gel Company, Inc., San Francisco, CA, USA). After the detection of chemiluminescence signal, PVDF membranes were treated with 0.2 N sodium hydroxide for 10 min at room temperature with gentle agitation to remove the antibodies and re-probed for another target protein. The levels of proteins were quantitated from the captured image using LI-COR Image Studio Lite Software 5.0 (LI-COR Biosciences, Lincoln, NE, USA) and normalized to the amount of GAPDH detected from the same PVDF membrane.

### Statistical Analysis

For quantitative results, the average value of each experimental group was calculated and compared using GraphPad Prism software version 8.01 (GraphPad Software, Inc., La Jolla, CA, USA). Statistical significance of the differences between sham and BLG groups was independently analyzed for male and female groups using unpaired *t*-tests. Welch's correction was used when sample sizes varied, and Mann-Whitney test was employed where normal distribution of data values was not observed. A *p*-value less than 0.05 (*p* < 0.05) was considered statistically significant.

## Results

### BLG Sensitization of C57BL/6J Mice Results in Increased Serum Levels of Allergen-Specific IgE and IgG1 in Male Mice Without Eliciting Obvious Signs of Anaphylaxis After BLG Challenge

**Figure 2:**
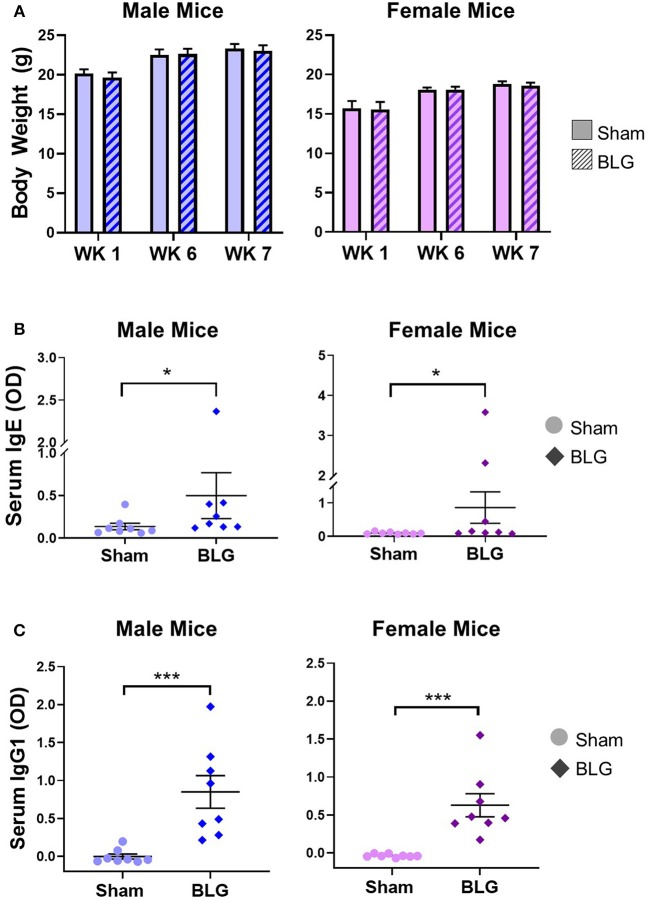
Physical growth and adaptive immunity development during BLG sensitization. **(A)** Weights of mice were recorded during Weeks 1, 6, and 7 of sensitization to assess potential impact of the sensitization regime on overall health and growth. **(B,C)** Serum isolated from the terminal blood was used to quantify the levels of BLG-specific IgE **(B)** or IgG1 **(C)** using ELISA. Values shown in the graphs indicate the group average ± SEM, *p < 0.05; ***p < 0.001. (Mann-Whitney test), male sham: *n* = 8; male BLG: *n* = 8; female sham: *n* = 8; female BLG: *n* = 8.

To monitor the overall health and steady growth of the experimental mice, their body weights were recorded during the sensitization protocol. The average body weights of mice in each group before the initiation of sensitization (Week 1) and at the time of allergen challenges (Week 6 and 7) were compared between the sex-matched treatment groups (Figure 2A). No significant differences were found in the body weights between the groups at any of the time points examined, suggesting that BLG-sensitized mice had comparable growth to the sham mice.

A week after the 5 weekly sensitization procedures, all mice underwent a BLG challenge in Week 6 for the assessment of their physical responses to allergen re-exposure. No obvious signs of anaphylaxis were exhibited by male or female mice from both of the treatment groups at 30 min post-challenge. This lack of physical reactions to the allergen was observed again after the second challenge in Week 7.

In order to ensure that the BLG-sensitization protocol successfully induced acquired immunity in our mouse model, we next determined the levels of serum BLG-specific IgE and IgG1 using ELISA (Figures 2B,C, respectively). While the serum levels of BLG-specific IgE were comparable among the male and female sham groups, both of the BLG sensitized groups showed wider ranges of BLG-specific IgE levels. The serum samples from a few mice in each group contained much greater BLG-specific IgE than the others within the group (Figure 2B). There were modest but significant increases in the average levels of BLG-specific IgE in both sensitized male and female mice compared to their respective sham groups (male sham: 0.14 ± 0.04; male BLG: 0.5 ± 0.3; female sham: 0.10 ± 0.01; female BLG: 0.9 ± 0.5; *n* = 8 in all groups, *p* < 0.05, Mann-Whitney test). When the extreme values were identified as outliers by GraphPad Prism software and removed from the analysis, the statistical significance of the BLG-induced IgE levels increased to *p* < 0.01 for male mice (male sham: 0.10 ± 0.02, *n* = 7; male BLG: 0.23 ± 0.05, *n* = 7; one outlier from each group was removed from the analysis [sham, 0.40; BLG, 2.37]). In contrast, removal of outliers from the female groups resulted in the loss of statistical significance for female groups [female sham: 0.10 ± 0.01, *n* = 8; female BLG: 0.17 ± 0.06, *n* = 6; two outliers removed from the analysis of the BLG group [3.58, 2.32]. The analysis excluding the outliers is shown in Supplementary Figure 1. Similarly, BLG-specific IgG1 levels were also elevated in the sensitized mice for both sexes with less variability than IgE (Figure 2C). These results indicated that the BLG-sensitization procedure elicited acquired immunity toward BLG with elevated antigen-specific IgE and IgG1 in both male and female mice. Although a subset of sensitized mice showed greater degrees of antibody productions, their apparent physical health was not visibly affected, and the allergen challenge did not result in anaphylaxis.

### BLG Sensitization Resulted in Anxiety- and Depression-Like Behavioral Changes in Male C57BL/6J Mice

**Figure 3:**
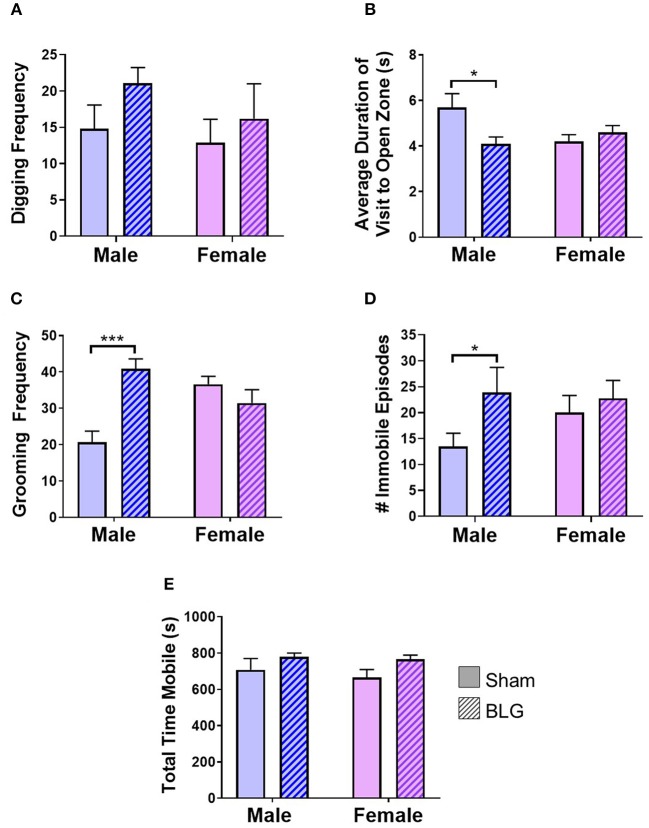
Assessments of anxiety- or depression-like behavior after BLG challenge. Behavioral tests were performed at 1- and 2-day post-challenge. **(A)** Digging frequency was quantified by two observers who were unaware of the experimental conditions. Either the presence (1 point) or absence (0 points) of digging behavior was recorded in every 10-s interval during the 10-min test period. The total points scored by the two observers were averaged and used as the final score for each mouse. **(B)** For EZM test, the average duration each mouse spent within the open zone per visit was computed by ANYmaze software and later validated by an observer. **(C)** Grooming frequency was quantified by two observers for scoring either the presence (1 point) or absence (0 points) of grooming behavior as described for the digging frequency scoring. **(D)** For TST, the number of immobility episodes was used as the measure of the mice's helpless behavior that reflected their depression-like state. **(E)** Total time mobile was also computed from the recordings during the digging behavior to verify their motility to distinguish their immobility from lethargy. Values shown in the bar graphs indicate the group average ± SEM, *p < 0.05, ***p < 0.001 (unpaired *t*-tests) *n* = 7–8.

BLG-mediated changes in mouse behaviors were examined using 4 different behavioral tests. The digging behavior observation and EZM were performed after the first challenge while grooming behavior observation and TST were carried out after the second challenge (see the Methods section). The frequency of digging activity within the 10-min observation period was 15 ± 3 in male sham, 21 ± 2 in male BLG-sensitized, 13 ± 3 in female sham, and 16 ± 5 in female BLG-sensitized mice (*n* = 8 per group, Figure 3A). Although there was a trend toward increased digging activity in male BLG-mice compared to their sex-matched sham, the difference did not reach a statistical significance (*p* = 0.1). However, the EZM showed a significant decrease in the average duration of visit to open zone in male mice (sham: 5.7 ± 0.6, *n* = 6; BLG: 4.1 ± 0.3; *n* = 7; *p* < 0.05) and not in female (sham 4.2 ± 0.3, *n* = 8; BLG: 4.6 ± 0.3; *n* = 7; Figure 3B). Further surveillance of the test recordings revealed that the mice with a shorter duration of visit to open zones often did not walk through the open zone to the other closed zone, but they briefly surveyed the entry to the open zone and returned to the original closed zone (see Supplementary Material). In addition, the analysis of grooming behavior after the second BLG challenge indicated that BLG-sensitized male mice groomed themselves more often than their sham counterpart (sham: 20 ± 3, *n* = 8; BLG: 41 ± 3; *n* = 8; *p* < 0.001) while no sensitization-dependent differences were observed between the female groups (sham: 36 ± 2, *n* = 8; BLG: 31 ± 4; *n* = 8), indicating that only male BLG-sensitized mice displayed more anxiety-like behavior than sham mice (Figure 3C). Similarly with the TST (Figure 3D), only male BLG-mice exhibited more depression-like behavior than the sham with greater numbers of immobile episodes during the 6-min testing period (sham: 14 ± 3, *n* = 8; BLG: 24 ± 5; *n* = 8; *p* < 0.05) while female sensitized mice did not (sham: 20 ± 3, *n* = 8; BLG: 23 ± 3; *n* = 8). To assure that the observed behavioral differences in the sensitized mice were not due to lethargy-related immobility, the total time in seconds mice were mobile during their digging tests were compared between the groups (Figure 3E). There were no obvious differences in the total time mobile among male and female sham and BLG mice, indicating that the changes in the behavioral parameters observed with the male BLG-sensitized mice did not result from physical or ambulatory difficulties. These results demonstrated that the male mice were susceptible to behavioral alterations upon BLG sensitization, even though they did not exhibit apparent anaphylactic reactions when challenged with the allergen.

### BLG Sensitization Altered the Levels of Tight Junction Protein and the Expression of Proinflammatory Cytokine in the Small Intestine

**Figure 4:**
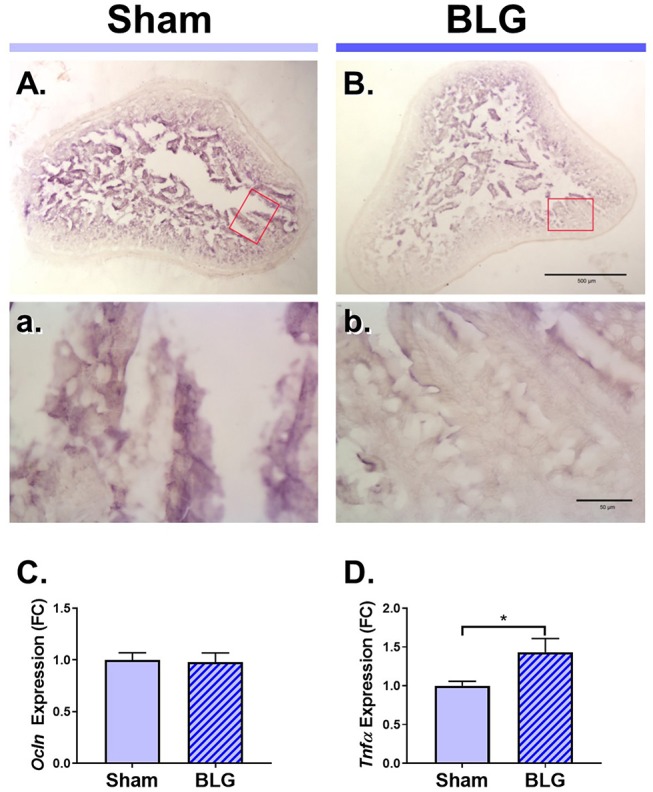
Immunohistochemical detection of occludin and the RT-qPCR assays for *Ocln* and *TNF*α expression in the ileum of male sham and BLG mice. Ileal sections (14 μm) from male sham **(A,a)** and BLG **(B,b)** mice were immunostained for occludin. The red rectangles in panels **(A,B)** indicate where the respective higher magnification images, **(a)** and **(b)**, were taken. The low-magnification **(A,B)** and high-magnification **(a,b)** images were taken with a 4X and 40X objectives, respectively. Scale bars: 500 μm for **(A)** and **(B)**; 50 μm for **(a)** and **(b)**. The expression levels of *Ocln* **(C)** and *Tnf*α **(D)** in the ileal tissue samples were also quantitated using RT-qPCR assay. The Cq values for *Ocln* and *Tnf*α were normalized to the Cq values of *Gapdh* (ΔCq) to calculate the expression values (ΔCq = 2^−Δ*Cq*^). Values shown in the bar graphs are expressed as the fold change (ΔΔCq) ± SEM. *p < 0.05 (unpaired *t*-test with Welch's correction), *n* = 6–8.

Next, we sought to identify changes in different brain regions that might contribute to the observed sex-dependent behavioral abnormality. Since BLG-mediated behavioral changes were only observed in male mice, we focused our further analyses on male animals. Allergens that paracellularly enter intestinal mucosa through compromised epithelial tight junction barriers may be recognized as pathogens by antigen presenting cells (APCs) and initiate immune responses. To examine whether BLG sensitization resulted in decreased barrier integrity, the levels of a tight junction protein, occludin, were examined in the intestinal tissue. Immunohistochemical assessment of the ileum from the male sham (Figures 4A,a) and BLG-sensitized mice (Figures 4B,b) showed that there was decreased staining in the villi of the latter group. This decreased immunoreactivity for occludin was likely a result of post-translational regulation since the amount of occludin transcripts in the ileal tissue from BLG-sensitized mice did not differ from the sham mice when determined using RT-qPCR (Figure 4C).

Aberrant paracellular infiltration of allergens could trigger inflammatory responses by intestinal immune cells. Thus, we also investigated the inflammatory status of the gut mucosa by determining the amount of a proinflammatory cytokine, TNFα. An RT-qPCR assay indicated that there was a modest but significant increase in TNFα mRNA in the ileum (Figure 4D), signifying the presence of proinflammatory events at the site of allergen insult.

### GFAP-Immunoreactive Astrocytes Were Hypertrophic in the Midbrain Region of the BLG-Sensitized Mice

**Figure 5:**
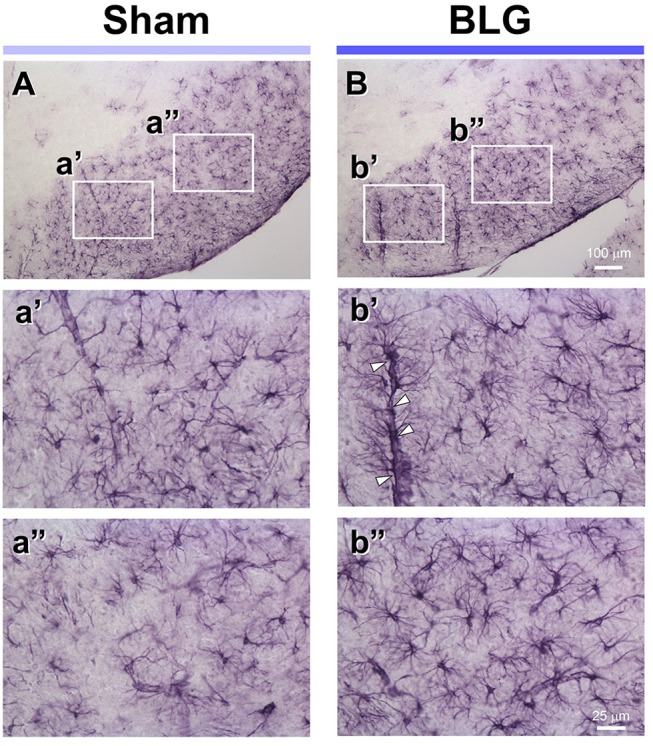
GFAP immunoreactivity in the midbrain of sham and BLG-sensitized mice. GFAP-positive astrocytes were identified by immunohistochemically staining brain sections (40 μm). Representative midbrain sections from sham **(A,a',a”)** and BLG-sensitized **(B,b',b”)** male mice are shown. The white rectangles in **(A,B)** indicate where the respective higher magnification images **a'–b”** were taken. The open arrowheads indicate dense GFAP-immunoreactive astrocyte end-feet along the blood vessel. The low-magnification **(A,B)** and high-magnification **(a',a”, b',b”)** images taken with a 10X and 40X objectives, respectively. Scale bars: 100 μm for **(A)** and **(B)**; 25 μm for **a'–b”**.

Under the hypothesis that glia cells could respond to inflammatory mediators from the intestine and elicit neuroinflammation that would ultimately result in altered behavior, brain tissues from sham and BLG-sensitized male mice were immunostained for astrocyte and microglia markers, GFAP and Iba1, respectively. Iba1-immunopositive cells were observed throughout the brain sections although we did not observe noticeable differences between sham and BLG-sensitized mice (not shown). GFAP-stained astrocytes were also found ubiquitously in the brain, but they were more localized to specific regions such as within the white matter. In midbrain sections, the majority of astrocytes were found within the substantia nigra pars reticulata (Figure 5). GFAP-positive cells were abundantly present in both sham (Figure 5A) and BLG-sensitized (Figure 5B) mice. Interestingly, astrocytes in this area of BLG-sensitized mouse brain appeared darker and their processes seemed greater in number and thickness (Figures 5b',b”). Perivascular astrocytes were notably different, with apparently increased density of GFAP-positive end-feet contacting the vascular wall (arrowheads in Figure 5b'). These observations provided evidence for glial response, at least by astrocytes in the midbrain regions, in the central nervous system of BLG-sensitized mice.

**Figure 6:**
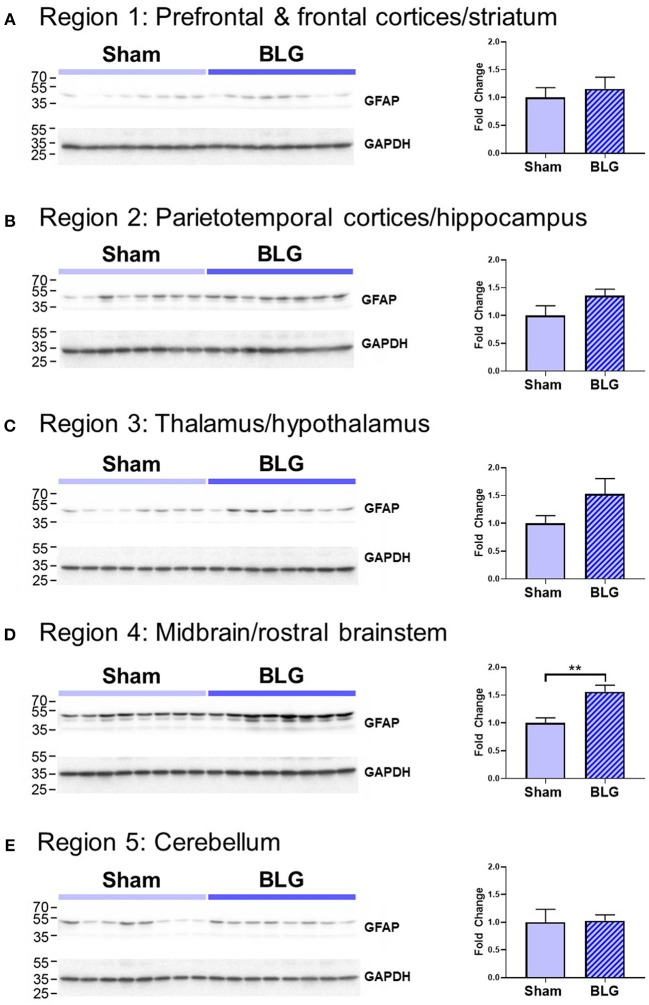
Western blot analysis of GFAP in the isolated five brain regions. Soluble proteins isolated from the 5 regions were resolved on discontinuous 15% SDS-polyacrylamide gels for western blot detection of GFAP (upper panels). **(A)** Region 1, **(B)** Region 2, **(C)** Region 3, **(D)** Region 4, and **(E)** Region 5 as described in Figure 1B. Chemiluminescence signals for GFAP were digitally captured and shown in the upper panels. GAPDH was also detected from the same blots and used as a reference for loading variability (lower panels). The captured GFAP signals were quantified using LI-COR Image Studio Lite software and normalized to GAPDH signals. Values shown in the bar graphs indicate the group average ± SEM. **p < 0.01 (unpaired *t*-test), *n* = 8.

**Figure 7:**
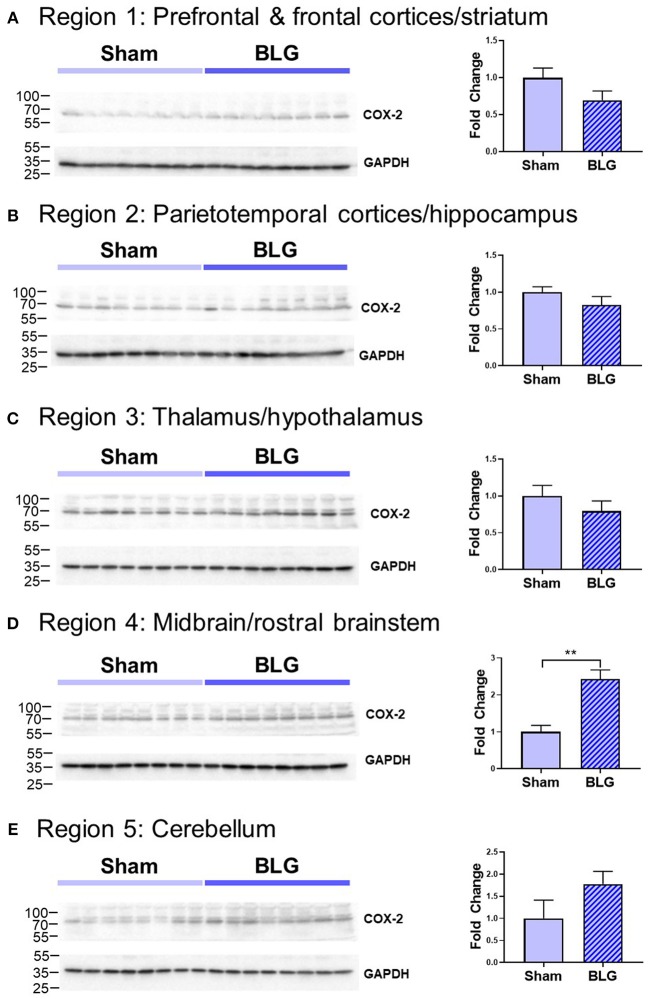
Western blot analysis of COX-2 in the isolated five brain regions. Soluble proteins isolated from the five regions were resolved on discontinuous 15% SDS-polyacrylamide gels for western blot detection of COX-2 (upper panels). **(A)** Region 1, **(B)** Region 2, **(C)** Region 3, **(D)** Region 4, and **(E)** Region 5 as described in Figure 1B. Chemiluminescence signals for COX-2 were digitally captured and shown in the upper panels. GAPDH was also detected from the same blots and used as a reference for loading variability (lower panels). The captured COX-2 signals were quantified using LI-COR Image Studio Lite software and normalized to GAPDH signals. Values shown in the bar graphs indicate the group average ± SEM. **p < 0.001 (unpaired *t*-test), *n* = 8.

In order to verify our immunohistological observations, western blot analysis was performed using protein extracts from different brain regions (Figure 6, see Figure 1B for the division of the regions). The level of GFAP was slightly elevated in the Region 2 (parietotemporal cortices and hippocampus) and Region 3 (thalamus and hypothalamus) of the BLG-sensitized mice, although the difference was not statistically significant (Region 2: 1.4 ± 0.2-fold, *p* = 0.1; Region 3: 1.5 ± 0.3-fold, *p* = 0.1). However, in Region 4 containing the midbrain and rostral brainstem, the difference in GFAP levels between the two groups of mice was significant with a 1.6 ± 0.2-fold increase in the sensitized mice (*p* < 0.001). This result indicated that BLG sensitization resulted in upregulation of GFAP in this region, corroborating our immunohistological observation of hypertrophic astrocytes in the same region. As an additional marker of proinflammatory change, we next examined protein levels of COX-2 in the various brain regions (Figure 7). Exactly as observed when examining GFAP levels, a significant increase in COX-2 protein levels in sensitized mouse brains was noted only in the midbrain and rostral brainstem samples (Region 4).

### The Proinflammatory Cytokine, TNFα, Was Elevated in the Midbrain Region

**Figure 8:**
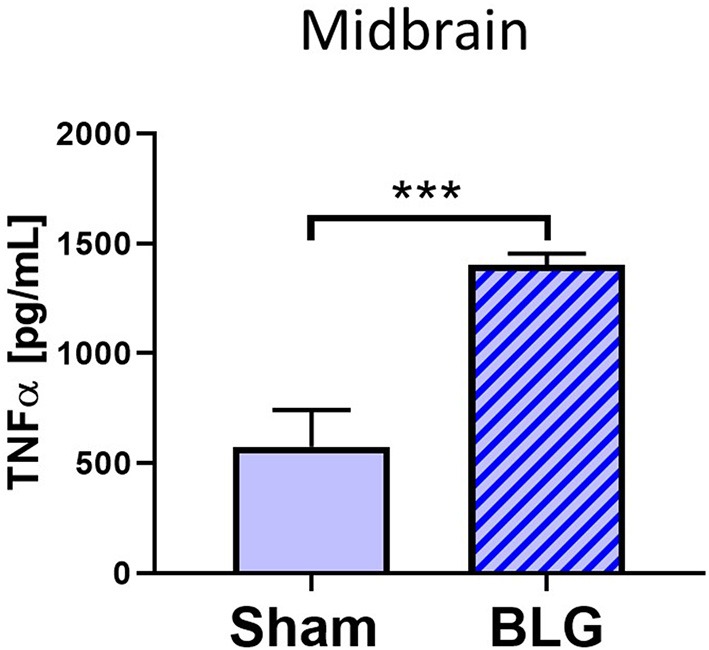
Quantification of TNFα levels in the midbrain region of sham and BLG male mice using ELISA. The levels of TNFα in the midbrain region were quantified by ELISA. Values indicate the group average ± SEM. ***p < 0.001 (unpaired *t*-test), *n* = 8.

Based on our observation of astrogliosis and elevated COX-2 protein levels in the midbrain regions, we hypothesized that the GFAP-positive reactive astrocytes might be responding to and/or producing inflammatory mediator(s). Since astrocytes are capable of producing and responding to TNFα (Eddleston and Mucke, 1993), we measured the levels of this proinflammatory cytokine in the midbrain region (Region 4) using ELISA (Figure 8). As predicted, the amount of TNFα was significantly elevated in this region of BLG-sensitized mice by ~2.7-fold (sham: 1,273 ± 384 pg/mL; BLG: 3,469 ± 194 pg/mL, *n* = 8). This result demonstrated that proinflammatory events are present at least in this region of the brain of BLG-sensitized mice and provided the evidence that sensitization to a milk allergen results in neuroinflammation associated with behavioral abnormality.

## Discussion

For several decades, the association of FAH with behavioral, emotional and cognitive impairments has been suggested, often referred to as “cerebral allergy” (Davison, 1949) or “allergic tension-fatigue syndrome” (Speer, 1954, 1958). More recently, a growing number of reports have more specifically described FAH comorbidities with depression (Patten and Williams, 2007; Garg and Silverberg, 2015; Ferro et al., 2016), anxiety (Lyons and Forde, 2004; Patten and Williams, 2007; Garg and Silverberg, 2014; Shanahan et al., 2014; Ferro et al., 2016), ADHD (Garg and Silverberg, 2014; Shanahan et al., 2014; Ferro et al., 2016; Topal et al., 2016), and autism (Lyall et al., 2015; Xu et al., 2018). However, the evidence that FAH in fact modifies physiological functions of the brain is still insufficient, and the mechanism remains to be elucidated.

One of the major obstacles in the assessment of brain pathophysiology in FAH-associated neuropsychiatric conditions is controlling the variables associated with the study subjects, such as genetic background, diet, socioeconomic status, local environment, and culture, all of which may contribute to differences in behavior as well as FAH development. In addition, experimental parameters for quantitative assessments are often limited to evaluation scores on questionnaires for neuropsychiatric conditions and blood IgE levels and/or skin tests for FAH. While it is undeniably challenging to evaluate mood- and emotion-elicited behavior in animal models, performing a series of behavioral tests helps to validate the results. Furthermore, animal models provide many advantages in an experimental study by allowing to control genetic, environmental, and dietary variables and to directly evaluate pathophysiology of the brain and other organs. Indeed, a mouse model of CMA with the C3H/HeOuJ strain has been utilized to demonstrate autistic-like deficit in social behavior and neurochemical changes in the brain (de Theije et al., 2014).

In the present study, we produced non-anaphylactic CMA in C57BL/6J mice using BLG as the allergen and assessed the cellular and molecular changes in the intestine and brain to identify CMA-induced pathology that might have contributed to their abnormal behavioral outcomes. During the 7 weeks of the sensitization/challenge period, both male and female mice showed no differences in their growth rate (Figure 2A). Importantly, we did not observe overt anaphylaxis symptoms in any of the groups after each of the two challenges at Week 6 and 7, although significant increases in BLG-specific IgE and IgG1 levels were observed in both male and female sensitized mice (Figures 2B,C). This result indicated that acquired immunity to BLG can be established without observable physical reactions.

Our behavioral assessments of sham and BLG-sensitized mice included digging and grooming frequencies, EZM, and TST (Figure 3). Digging behavior in rodents is an innate burrowing behavior, and the test is often performed by placing the animal in a cage with a thick layer of bedding without or with marbles (Deacon, 2006). Unlike the WP-sensitized mice we previously described (Germundson et al., 2018), BLG-sensitized mice did not exhibit decreased digging behavior. Instead, there was an increased trend in male sensitized mice, suggesting that the behavioral effect of BLG sensitization appears to be distinct from that of the WP mixture. Although the reason for the discrepancy between the two mouse models of CMA in this behavioral outcome is not clear, it may be postulated that other constituents in the WP mixture, such as α-lactalbumin, immunoglobulins, and lactoferrin (Farrell et al., 2004), had a more diverse effect than BLG alone.

Grooming is another intrinsic rodent behavior consisting of a complex series of movements, and the frequency, total time spent, and sequence of grooming can be affected by extrinsic factors such as stress (Kalueff et al., 2016). We observed that female sham mice groomed more frequently than their male counterpart (Figure 3C), and the frequency of female grooming behavior was not affected by BLG sensitization. On the contrary, BLG-sensitized male mice showed significantly elevated grooming behavior, indicative of their stressed or anxious state (Kalueff et al., 2016). This observation was corroborated by their performance in the EZM test, in which male BLG-sensitized mice spent significantly less time than the sham mice in the open zones when entered (Figure 3B). These results together support the notion that BLG-sensitized male mice exhibited anxiety-like behavior.

Because anxiety and depression are often comorbid (Johansson et al., 2013; Tiller, 2013), we also examined whether the BLG-sensitized mice would exhibit depression-like behavior. In the TST, depression-like behavior is quantified by the animal's immobility, which reflects decreased attempts to escape from the helpless position (Cryan et al., 2005). Our results demonstrated that male BLG-sensitized mice indeed displayed depression-like behavior, although no difference was observed between sham and BLG-sensitized female mice (Figure 3D). From our observation that the overall activity of male and female mice did not differ between sham and BLG-sensitized groups, it is unlikely that the immobility resulted from inability to move or lethargy (Figure 3E). Taken together, our behavioral tests indicated that BLG sensitization elicited anxiety- and depression-like behavior in male-specific manner. This sex-dependent behavior manifestation was also observed with WP-sensitized mice and have been discussed previously (Germundson et al., 2018).

Interestingly, similar sex differences in behavioral observations have been reported with human patients with neuropsychiatric disorders, including ADHD, obsessive-compulsive disorder (OCD), and autism spectrum disorder (ASD). Several meta-analysis studies have indeed found greater prevalence in male population (Hanna, 1995; Gaub and Carlson, 1997; Gershon, 2002; de Mathis et al., 2011; Russell et al., 2011). This male dominance in these conditions seem to arise from the fact that male patients exhibit more noticeable behavioral phenotypes than female patients. For example, boys with ADHD display more externalized and/or disruptive behavior than girls, who in contrast tend to show more internalized, inattentive behavior (Gaub and Carlson, 1997; Gershon, 2002). Similarly, some studies on sexual dimorphism in ASD symptomatology reported that boys have more severe autistic traits and therefore are more likely to be diagnosed with ASD than girls (Russell et al., 2011; Mandy et al., 2012). Biological factors, such as sex hormone-dependent structural development of the prefrontal and orbitofrontal cortices, thalamus, and basal ganglia (Maia et al., 2008), the volume of the pituitary gland (MacMaster et al., 2006), and polymorphisms in the serotonergic system (de Mathis et al., 2011; Verma et al., 2014; Shuffrey et al., 2017), have been suggested to underlie the sex differences in behavioral manifestations.

In addition to behavioral differences, sexual dimorphism of the immune systems has been well-recognized. Gene regulation by gonadal hormones and the expression of X-chromosome genes are known to differentially affect the immune system in males and females, including immunoglobulin productions, T-lymphocyte functions and allergic/atopic disease susceptibility and symptom severity [see reviews by DunnGalvin et al., 2006; Pennell et al., 2012; Klein and Flanagan, 2016]. However, male dominance of food allergy appears to be inconsistent across studies, depending on allergen types and patient age groups, as well as on the study method used and year examined (Jarvis and Burney, 1998; Becklake and Kauffmann, 1999; Kelly and Gangur, 2009; Acker et al., 2017). Therefore, the roles of these biological and immune dimorphisms in the sex-specific behavioral response to BLG sensitization and challenge are complex and require further scrutiny in humans as well as in our animal models of CMA.

Moving forward, we focused our pathophysiological investigation on male mice to assess histological and biochemical changes that might reflect the behavioral changes that deviated from the sham control. In orally-sensitized mice, the site of allergen insult is the gastrointestinal (GI) tract. Decreased mucosal occludin immunoreactivity and increased proinflammatory cytokine expression in the BLG-sensitized ileum suggested that the immune responses to the allergen during sensitization had impaired intestinal barrier (Figure 4). In addition, it is possible that dysbiosis had occurred during the sensitization and elicited these changes in intestinal physiology, since gut microbe compositions can be influenced by diet and shifts in compositions can result in inflammation (Round and Mazmanian, 2009; Clements and Carding, 2018). These changes in the gut physiology and microbiota are likely to be produced gradually during the sensitization period, rather than immediately after the BLG challenge, since immune activation status in food-allergen sensitized mice has been reported to be heightened as evidenced by greater proliferative capacity of splenocytes compared to naïve mice without restimulation with the allergen (Li et al., 2000). However, time course of pathophysiology development and potential involvement of gut microbiota are yet to be determined in our mouse model. Loss of intestinal barrier and intestinal dysbiosis have been reported in autistic patients (de Magistris et al., 2010; Fiorentino et al., 2016) and their implication in pathogenesis of neuropsychiatric conditions has been reviewed in recent literature (Karakula-Juchnowicz et al., 2016;Grochowska et al., 2018).

Inflammatory responses were also found in the brain of BLG-sensitized mice. Although we did not detect apparent microgliosis by Iba1 immunostaining (not shown), we observed notable differences in GFAP-positive astrocyte morphology in certain areas of the brain, especially perivascular regions of the midbrain (Figure 5). Interestingly, similar observations of hypertrophic perivascular astrocytes have been observed in our WP-sensitized aged mice (Germundson et al., 2018) and also reported in the spinal cord of EAE mice (Voskuhl et al., 2009). These astrocytes resemble scar forming astrocytes often described in central nervous system injuries and are thought to establish barriers to control infiltration of leukocytes from the blood circulation (Voskuhl et al., 2009; Sofroniew and Vinters, 2010). Importantly, increased expression of GFAP plays a crucial role in this barrier formation since ablation of GFAP-expressing astrocytes results in profound increases in the number of leukocyte infiltrates in the spinal cord of EAE mice (Voskuhl et al., 2009). Thus, it is feasible to postulate that BLG sensitization stimulated peripheral immune cells and increased their circulating levels, and the perivascular astrocytes had become activated to regulate the amount of inflammatory influence from the periphery. To provide evidence for this notion, juxtaposition of leukocytes with GFAP-immunoreactive astrocyte end-feet across the blood vessel walls need to be demonstrated in our mouse model of CMA as shown in the EAE mice (Voskuhl et al., 2009). Nonetheless, semi-quantitative analysis with western blotting showed that the GFAP levels in the midbrain regions were significantly elevated in BLG-sensitized mice when compared to sham mice (Figure 6), supporting our immunohistochemical observations.

Astrocytes are multifaceted glia cells in the central nervous system, and they play essential roles in metabolic support, intercellular signaling, blood flow regulation, myelination, and synaptic pruning [reviewed by Sofroniew and Vinters, 2010]. It is of interest to examine whether these functions of astrocytes become dysregulated in BLG-sensitized mice and influence their behavior. Astrocytes are also important mediators of neuroinflammation with the ability to produce and secrete pro- as well as anti-inflammatory molecules (Eddleston and Mucke, 1993; John et al., 2003). The fact that the levels of TNFα were significantly elevated in the midbrain regions of the BLG-sensitized mice suggested that the astrocytes were acting as proinflammatory mediators (Figure 7). However, it seems counterintuitive that microglia did not show reactive morphology in response to the elevated proinflammatory cytokine levels. One possible explanation is that TNFα detected in our samples had derived from the intestines or circulating leukocytes and was not produced by astrocytes, which had successfully prevented the cytokine and cytokine-producing cells from activating microglia. An alternative explanation may be that our experimental paradigm was too transient, and BLG-sensitized mice needed to be repetitively challenged to elicit more chronic inflammation in order for microglia to become activated. These hypotheses, along with the possible involvement of other proinflammatory cytokines, such as IL-1β and IL-6, need to be tested in future studies.

In conclusion, we have demonstrated that sensitization of C57BL/6J mice with BLG induces anxiety- and depression-like behaviors in male mice that are associated with decreases in tight junction proteins in the intestines and astrogliosis in the brain. Elevated TNFα levels in both of these locations suggest that this proinflammatory cytokine plays a role, at least in part, in mediating immune responses to the cow's milk allergen in sensitized mice. Whether these pathophysiological findings directly influence the behavior of sensitized mice is yet to be determined. However, clinical reports of symptom improvements in patients with treatment-resistant depression and other psychiatric conditions after elimination diet (Parker and Watkins, 2002) and plasmapheresis (Barzman et al., 2018) support the involvement of FAH-triggered immune responses in pathogenesis of behavioral disorders. Treatments with antihistamines and/or steroidal/non-steroidal anti-inflammatory reagents to, respectively, inhibit the effects of hypersensitivity-mediated immediate immune reactions (e.g., mast cell degranulation) and subsequent inflammation in our mouse model will be useful in clarifying the involvement of proinflammatory cytokines in the development of observed brain pathophysiology and behavioral changes. Elucidating the mechanisms by which immune responses to a dietary component manifest as brain and behavioral dysfunction may therefore provide potential therapeutic approaches beyond the use of neuromodulatory drugs.

## Data Availability

All datasets generated for this study are included in the manuscript and/or the Supplementary Files.

## Ethics Statement

This study was carried out in accordance with the recommendations of University of North Dakota Institutional Animal Care and Use Committee. The protocol was approved by the University of North Dakota Institutional Animal Care and Use Committee.

## Author Contributions

NS performed the experiments, data collection and analyses, and drafted the manuscript. DG and CC assisted with the experiments, data and tissue collection and analyses, and critically reviewed and edited the manuscript. LV assisted with the experiments and data collection. KN-C designed, planned, and performed the experiments and analyses, drafted and edited the manuscript, and obtained the funding for the study. This work, including the purchases of the animals and reagents, was supported by an Institutional Development Award (IDeA) from the National Institute of General Medical Sciences of the National Institutes of Health (P20GM103442), University of North Dakota Epigenomics of Development and Disease Center of Biomedical Research Excellence Pilot Grant (5P20GM104360-05).

### Conflict of Interest Statement

The authors declare that the research was conducted in the absence of any commercial or financial relationships that could be construed as a potential conflict of interest.

## Footnotes

^1^National Institute of Mental Health. Center for Behavioral Health Statistics and Quality (2017). Retrieved from https://www.nimh.nih.gov/health/statistics/mental-illness.shtml.

## Supplementary Material

Video S1 Male Sham mouse carefully walking across the open zone of the EZM.

Video S2 Male BLG mouse briefly surveying the open zone of the EZM and returning to the closed zone.

Supplementary Figure 1 An alternative analysis for the post-sensitization serum levels of BLG-specific IgE shown in Figure 2B. Serum isolated from the terminal blood was used to quantify the levels of BLG-specific IgE using ELISA. A group analysis including all sample values are shown in Figure 2B. As an alternative analysis of the results, outliers within each group were identified using GraphPad Prism software (ROUT, Q = 1%), and Mann-Whitney test was performed excluding the outlier values from the statistical analysis. For male groups, statistical significance of ^**^*p* < 0.01 was found between sham and BLG mice (male sham: 0.10 ± 0.02, *n* = 7; male BLG: 0.23 ± 0.05, *n* = 7; one outlier from each group was removed from the analysis [sham, 0.40; BLG, 2.37]). Statistically significant difference between female sham and BLG groups was not found using this method of analysis [female sham: 0.10 ± 0.01, *n* = 8; female BLG: 0.17 ± 0.06, *n* = 6; two outliers removed from the analysis of the BLG group [3.58, 2.32].
