# Supplementary figures and images for: Astrogliosis Associated With Behavioral Abnormality in a Non-anaphylactic Mouse Model of Cow's Milk Allergy
Source: Front Cell Neurosci. 2019 Jul 16;13:320. doi: 10.3389/fncel.2019.00320 (PMC6646667; doi:10.3389/fncel.2019.00320)

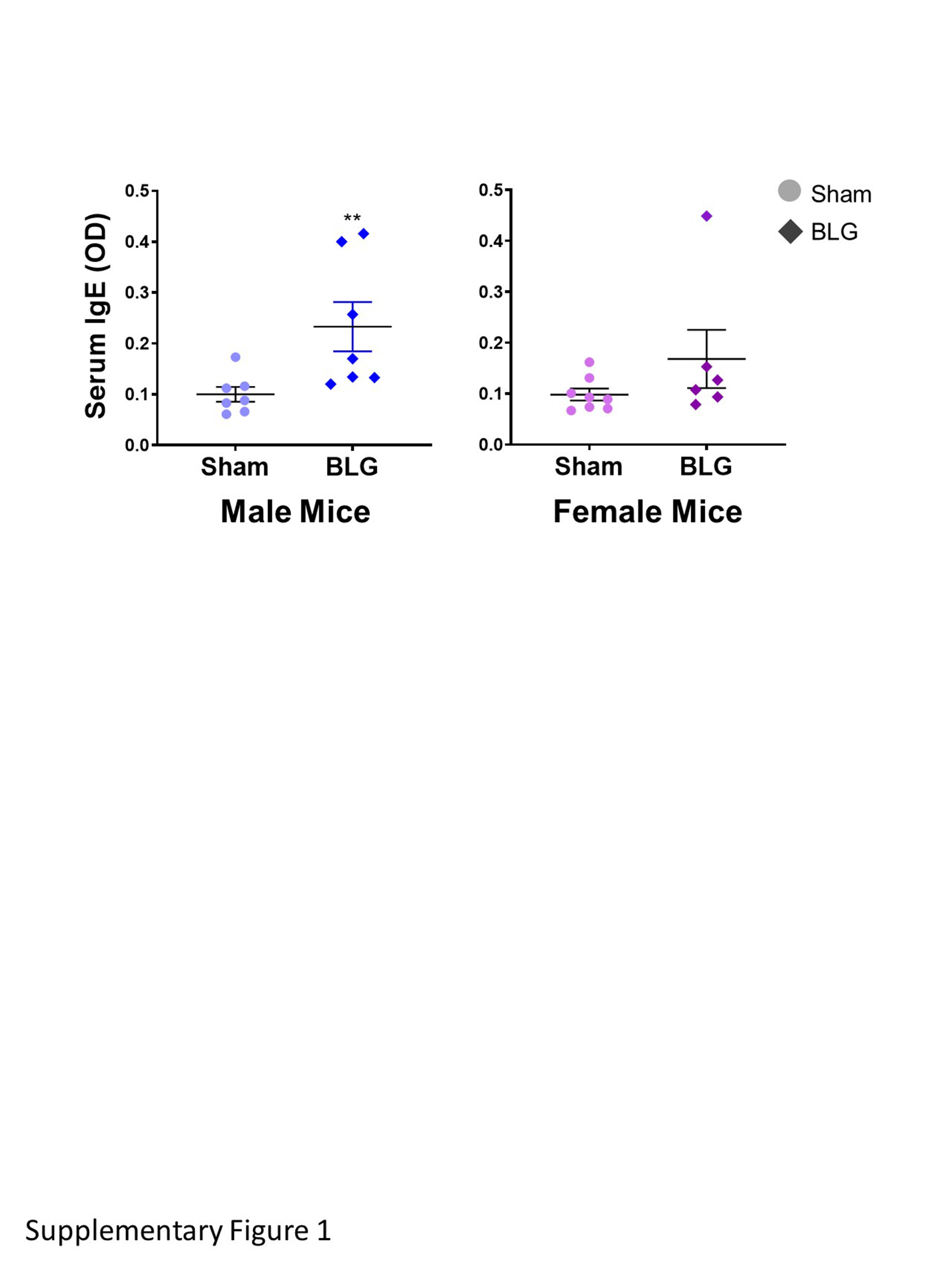

Supplement: Supplementary Figure 1 — An alternative analysis for the post-sensitization serum levels of BLG-specific IgE shown in Figure 2B. Serum isolated from the terminal blood was used to quantify the levels of BLG-specific IgE using ELISA. A group analysis including all sample values are shown in Figure 2B. As an alternative analysis of the results, outliers within each group were identified using GraphPad Prism software (ROUT, Q = 1%), and Mann-Whitney test was performed excluding the outlier values from the statistical analysis. For male groups, statistical significance of **p < 0.01 was found between sham and BLG mice (male sham: 0.10 ± 0.02, n = 7; male BLG: 0.23 ± 0.05, n = 7; one outlier from each group was removed from the analysis [sham, 0.40; BLG, 2.37]). Statistically significant difference between female sham and BLG groups was not found using this method of analysis [female sham: 0.10 ± 0.01, n = 8; female BLG: 0.17 ± 0.06, n = 6; two outliers removed from the analysis of the BLG group [3.58, 2.32]. [file Image_1.TIF]
